# Two-in-one: UV radiation simultaneously induces apoptosis and NETosis

**DOI:** 10.1038/s41420-018-0048-3

**Published:** 2018-04-27

**Authors:** Dhia Azzouz, Meraj A. Khan, Neil Sweezey, Nades Palaniyar

**Affiliations:** 1 Program in Translational Medicine, Peter Gilgan Centre for Research and Learning, The Hospital for Sick Children, Toronto, ON Canada; 2 Department of Laboratory Medicine and Pathobiology, University of Toronto, Toronto, ON Canada; 3 Department of Paediatrics and Physiology, University of Toronto, Toronto, ON Canada; 4 Institute of Medical Sciences, Faculty of Medicine, University of Toronto, Toronto, ON Canada; 5 Massachusetts General Hospital and Shriners Hospitals for Children in Boston, Harvard Medical School, Boston, MA USA

## Abstract

NETosis is a unique form of neutrophil death that differs from apoptosis and necrosis. However, whether NETosis and apoptosis can occur simultaneously in the same neutrophil is unknown. In this paper, we show that increasing doses of ultraviolet (UV) irradiation increases NETosis, which is confirmed by myeloperoxidase colocalisation to neutrophil extracellular DNA. Increasing UV irradiation increases caspase 3 activation, mitochondrial reactive oxygen species (ROS) generation and p38, but not ERK, phosphorylation. Inhibition of mitochondrial ROS production and p38 activation, but not NADPH oxidase (NOX) activity, suppresses UV-induced NETosis, indicating that UV induces NOX-independent NETosis. Like classical NOX-dependent and -independent NETosis, UV-induced NETosis requires transcriptional firing for chromatin decondensation. Cell death-specific inhibitor studies indicate that UV-mediated NETosis is not apoptosis, necrosis or necroptosis. Collectively, these studies indicate that increasing doses of UV irradiation induce both apoptosis and NETosis simultaneously, but the ultimate outcome is the induction of a novel form of NOX-independent NETosis, or “ApoNETosis”.

## Significance

During infection, activation of neutrophil NADPH oxidase leads to the generation of neutrophil extracellular traps (NETs) that could trap microbes. However, NET formation (NETosis) during sterile inflammation is not well understood. NETosis is a unique form of cell death, different from apoptosis and necrosis. Here we show that higher doses of UV irradiation induce both apoptosis and NETosis at the same time in the same cell. This novel form of NETosis is independent of NADPH oxidase activation, but requires mitochondrial reactive oxygen species generation and p38 activation. UV-induced NETosis does not induce citrullination of histone but requires DNA metabolism. Understanding this novel form of ApoNETosis could help to explain UV irradiation-related inflammation. UV-induced NETs could also be used for NET clearance studies without the worry about chemicals, toxins or cytokines that are commonly used for inducing NETosis.

## Introduction

NETosis is a novel and distinct form of neutrophil death that results in the formation and release of neutrophil extracellular traps (NETs)^1–6^. NETs are decondensed chromatin decorated with cytotoxic components such as myeloperoxidase (MPO)^7,8^. NETs have also been reported to originate from neutrophil mitochondria^9^. Although NETosis may be beneficial during infection-related inflammation^10,11^, excess NET formation, particularly during sterile inflammation, can damage tissue and organs^4,12–14^ and has been implicated in many disease states^15–17^. Therefore, understanding the molecular mechanisms of various forms of NETosis is important for regulating unwanted NET formation. The molecular mechanism of NETosis, particularly when induced by irradiation, has not been elucidated. To date, two major types of NETosis have been described: NADPH oxidase (NOX)-dependent NETosis and NOX-independent NETosis^18–20^. In NOX-dependent NETosis, activation of NOX results in increased intracellular reactive oxygen species (ROS) formation, phosphorylation of mitogen-activated protein kinases (MAPKs; extracellular signal-regulated kinase (ERK), p38, c-Jun N-terminal kinase (JNK)), transcriptional firing, chromatin decondensation and ultimately NET release^21–24^. During classical calcium ionophore-induced NOX-independent NETosis, increased intracellular calcium allows the translocation of peptidylarginine deiminase 4 (PAD4), which citrullinates histones at promoter regions^18,25^. Mitochondrial ROS production and subsequent phosphorylation of specific MAPKs (e.g., p38) promote the transcriptional firing that is necessary for chromatin decondensation and NET release^24^. However, whether NETosis could take place concomitantly with other classical forms of cell deaths such as apoptosis is unknown.

Induction of apoptosis by ultraviolet (UV) irradiation has been studied in detail, and the signalling steps involved in this pathway are well characterised^26–29^. However, whether UV radiation can regulate NETosis is unknown. In the present study, we investigated the ability of UV to induce NETosis, and showed that it represents a novel form of NETosis. The knowledge gained from this study could help to understand the sterile inflammation that takes place during extended exposure to UV light or UV-based treatment strategies.

## Results

### UV induces NETosis in a dose-dependent manner with a profile similar to that of NOX-independent NETosis

**Fig. 1:**
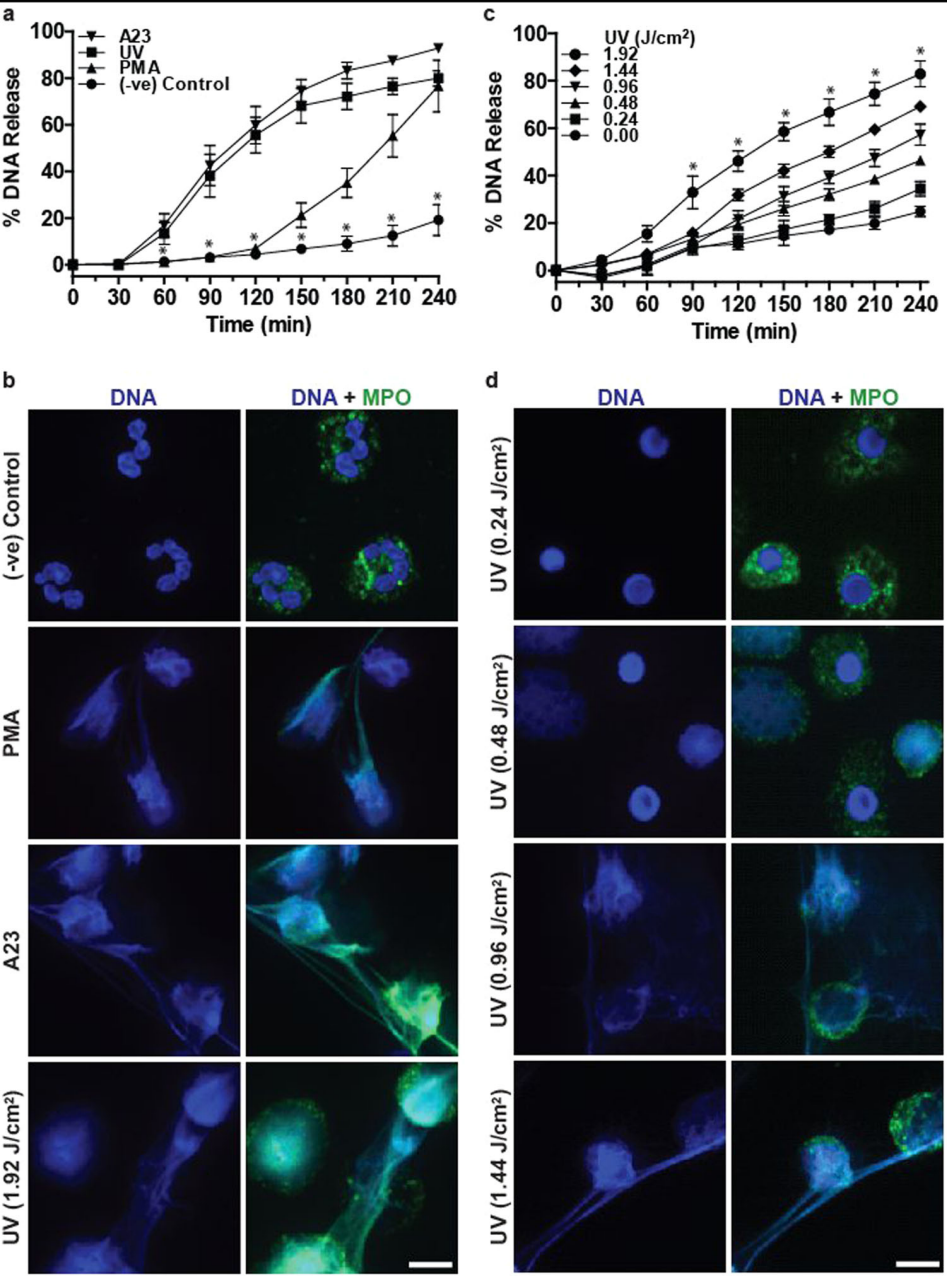
UV induces rapid NETosis and increasing UV dose increases NETosis. **a** DNA release from neutrophils following media (−ve control), PMA (25 nM) and A23 (4 µM) treatment or UV irradiation (1.92 J/cm^2^) was measured using the SYTOX Green plate reader assay. UV-induced NETosis follows the kinetics similar to that of A23-induced NETosis (*n* = 3; error bars represent SEM; A23, A23187; *p < 0.05). **b** Neutrophils were treated with PMA (25 nM), A23 (4 µM) or UV (1.92 J/cm^2^) and incubated for 240 min. Cells were stained for DNA (DAPI, blue) and MPO (green). Immunofluorescence imaging shows that MPO colocalises to DNA, hence 1.92 J/cm^2^ UV induces NETosis. Images are representative of three independent experiments. Scale bar, 10 μm. See low-magnification images in Fig. S2. **c** DNA release following treatment with increasing dose of UV was measured using the SYTOX Green plate reader assay (*n* = 3; error bars represent SEM; *p < 0.05). Increasing dose of UV results in increased NETosis. **d** Neutrophils were treated with varying doses of UV and incubated for 240 min. Cells were stained for DNA (DAPI, blue) and MPO (green). Confocal fluorescence imaging shows that UV light exposure results in NETosis at higher doses and apoptosis at lower doses. Images are representative of three independent experiments. Scale bar, 10 μm. See low-magnification images in Fig. S3

Short bursts of UV exposure induce apoptosis^28^. To determine whether high-dose UV irradiation could induce NETosis, we performed a SYTOX Green plate reader assay. SYTOX Green is a cell-impermeable dye that fluoresces green upon binding to DNA released by neutrophils; the amount of green fluorescence signal of this probe acts as a measure of NETosis. Time course data indicated that the kinetics of UV-induced NETosis was similar to the kinetics of NOX-independent NETosis (e.g., in response to the calcium ionophore A23187; hereafter referred to as A23) and not to NOX-dependent NETosis (e.g., phorbol 12-myristate 13-acetate (PMA); Fig. 1a). To confirm that the SYTOX Green assay data represent NETosis, cells were fixed at 120 or 240 min post stimulation, and stained for DNA with 4',6-diamidino-2-phenylindole (DAPI) and immunostained for MPO with fluorescently labelled antibodies. Confocal fluorescence images showed that MPO (green) decorated the DNA (DAPI, blue), both within the decondensing nuclei and on the extracellular net-like structures, confirming the induction of NETosis following PMA, A23 and 1.92 J/cm^2^ UV exposure (Fig. 1b). Images also confirmed the differences in SYTOX Green fluorescence kinetics. At this time point, chromatin was decondensed in PMA-treated neutrophils. By contrast, UV radiation rapidly induced NETosis and released NETs, similar to the kinetics of A23-induced NETosis (Fig. 1a, b, S2). Therefore, high-dose UV irradiation induces a rapid form of NETosis.

To determine the amount of UV irradiation required to induce NETosis, we exposed neutrophils to different doses of UV light, and conducted a SYTOX Green plate reader assay. UV dose-dependently induced DNA release (Fig. 1c). The regression analysis showed a typical saturation curve (*y* = 38.70*x* *−* 5.267*x*^2^ + 25.93; *r*^2^ = 0.9324), and SYTOX Green fluorescence levels started to plateau at high doses of UV light (0.96–1.92 J/cm^2^; Fig. S1). To confirm the induction of NETosis at different doses of UV, neutrophils were exposed to UV light (0.24–1.92 J/cm^2^), incubated for 240 min, fixed and stained for DNA and MPO. Confocal fluorescence microscopy indicated that at low doses (0.24–0.48 J/cm^2^) of UV irradiation, DNA (blue) and MPO (green) were present mainly in distinct compartments (nucleus vs. cytoplasm). By contrast, with increasing doses (0.96–1.92 J/cm^2^) of UV irradiation, MPO colocalised with DNA present within the decondensing nuclei and extracellular traps in most of the dying cells, confirming the induction of NETosis following the exposure of neutrophils to high-dose UV exposure (Fig. 1d, S2; S3). Taken together, these results show that UV irradiation of neutrophils induces NETosis in a dose-dependent manner, and extensive NETosis occurs at higher doses of UV irradiation.

### UV irradiation simultaneously induces both NETosis and apoptosis

**Fig. 2:**
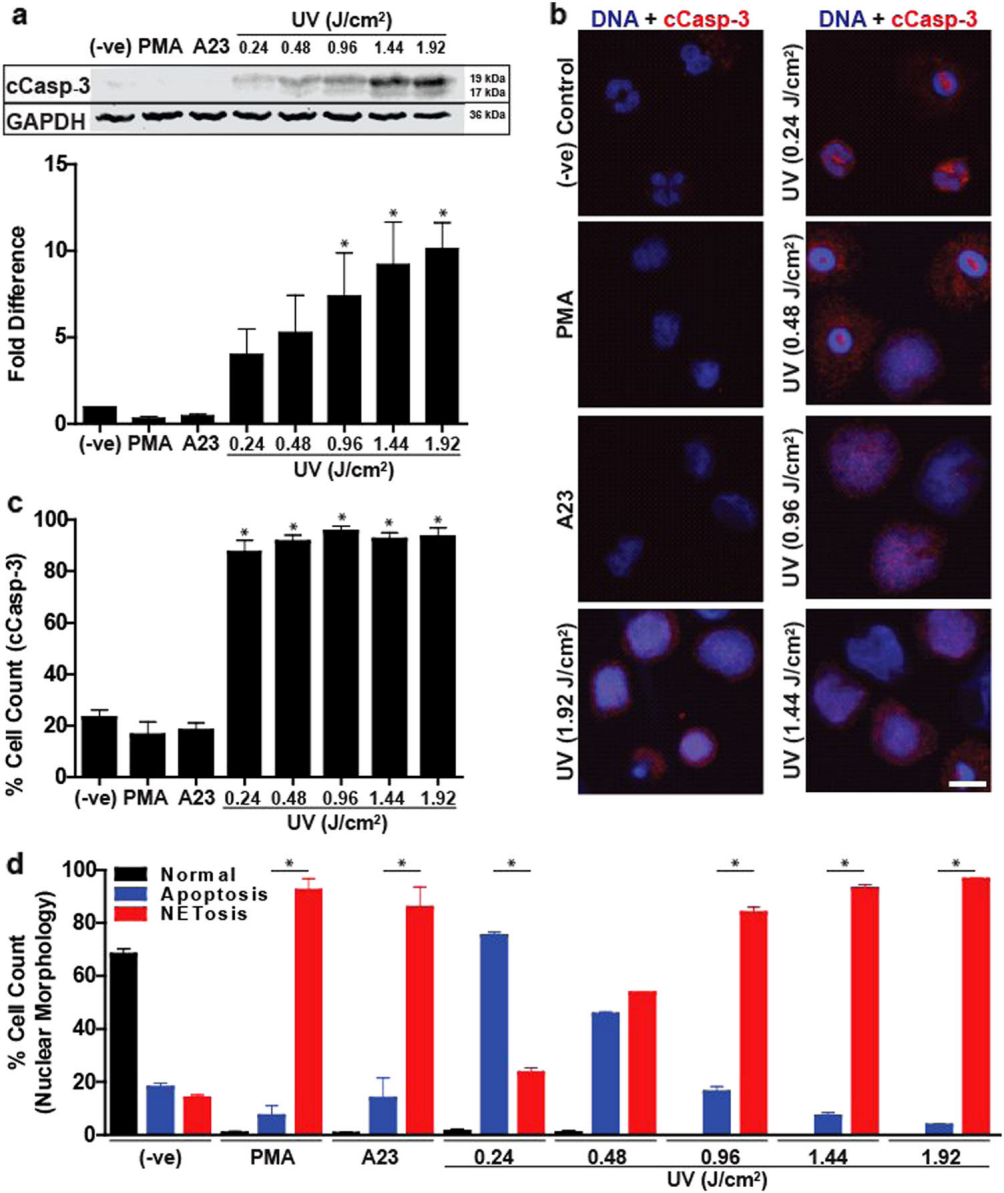
Increasing UV irradiation increases caspase 3 cleavage and DNA decondensation. **a** Human neutrophils were collected 2 h after activation with PMA (25 nM), A23 (4 µM) or increasing doses of UV. Immunoblots show that cleaved caspase 3 is found only in the UV conditions, and increase in UV doses increases caspase 3 cleavage (activation). Densitometry analysis confirms that caspase 3 cleavage dose-dependently increases following increased UV treatment (*n* = 3; *p < 0.05 for comparing with the negative control). **b** Neutrophils were treated with PMA (25 nM), A23 (4 µM) or varying doses of UV and incubated for 120 min. Cells were stained for DNA (DAPI, blue) and cleaved caspase 3 (red). Immunofluorescence imaging shows that cleaved caspase 3 is found in cells irradiated with UV light (scale bar, 10 μm). **c** Percentage of cells containing cleaved caspase 3 in cells treated with PMA (25 nM), A23 (4 µM) or varying doses of UV was calculated by counting cells immunostained for cleaved caspase 3 (n = 3; *p < 0.05 for comparing with the negative control). **d** Percentage of cells undergoing apoptosis, NETosis or neither (determined by nuclear morphology) in cells treated with PMA (25 nM), A23 (4 µM) or varying doses of UV was calculated by counting cells stained for DNA after 240 min (*n* = 3, *p < 0.05 for comparing percentage undergoing NETosis to percentage undergoing apoptosis within each treatment). Images are representative of three independent experiments. Error bars represent SEM; A23, A23187. See low-magnification images in Fig. S4

The hallmarks of UV-induced apoptosis are the cleavage of caspase 3 and condensation of nuclei^30–32^. Therefore, to determine the status of apoptosis in neutrophils following exposure to different doses of UV irradiation, caspase 3 cleavage was determined in these cells at 120 min time point by western blotting and immunocytochemistry, before most of the cells started to release NETs. Western blotting showed no increase in caspase 3 cleavage during PMA- or A23-induced NETosis. By contrast, caspase 3 cleavage increased with increasing doses of UV irradiation (Fig. 2a). To identify whether increasing doses of UV irradiation increased caspase 3 cleavage in more cells, we conducted immunocytochemistry. Confocal microscopy showed that caspase 3 was cleaved in most of the cells, but appeared as punctate dots in the cytoplasm of neutrophils at low-dose UV irradiation (0.24 and 0.48 J/cm^2^). By contrast, at higher doses of UV irradiation, cleaved caspase 3 was distributed throughout the cells (Fig. 2b, S4). Manual counting of cells showed that regardless of the UV dose, caspase 3 cleavage occurred in more than 87% of the neutrophils irradiated with UV light (Fig. 2c). Therefore, all of the doses of UV tested in the study induced apoptosis in neutrophils. Next, we quantified the cells using typical apoptotic (condensed) and NETotic (decondensed) nuclear morphology at 240 min, a time point in which most of the neutrophils underwent PMA-mediated and 1.92 J/cm^2^ UV-mediated NETosis. The majority of cells (76%) treated with low-dose UV (0.24 J/cm^2^) displayed apoptotic nuclear morphology, whereas cells treated with high doses of UV (0.96–1.92 J/cm^2^) displayed mostly (84–96%) NETotic nuclear morphology (Fig. 2d). With increasing doses of UV irradiation, the relative proportion of NETotic nuclei increased while apoptotic nuclei decreased. These results (Fig. 2) show that higher doses of UV irradiation simultaneously induce both apoptosis and NETosis in the same cells, and at high UV doses NETosis predominates in these neutrophils.

### Mitochondrial ROS, but not NOX-mediated ROS, is necessary for UV-induced NETosis

**Fig. 3:**
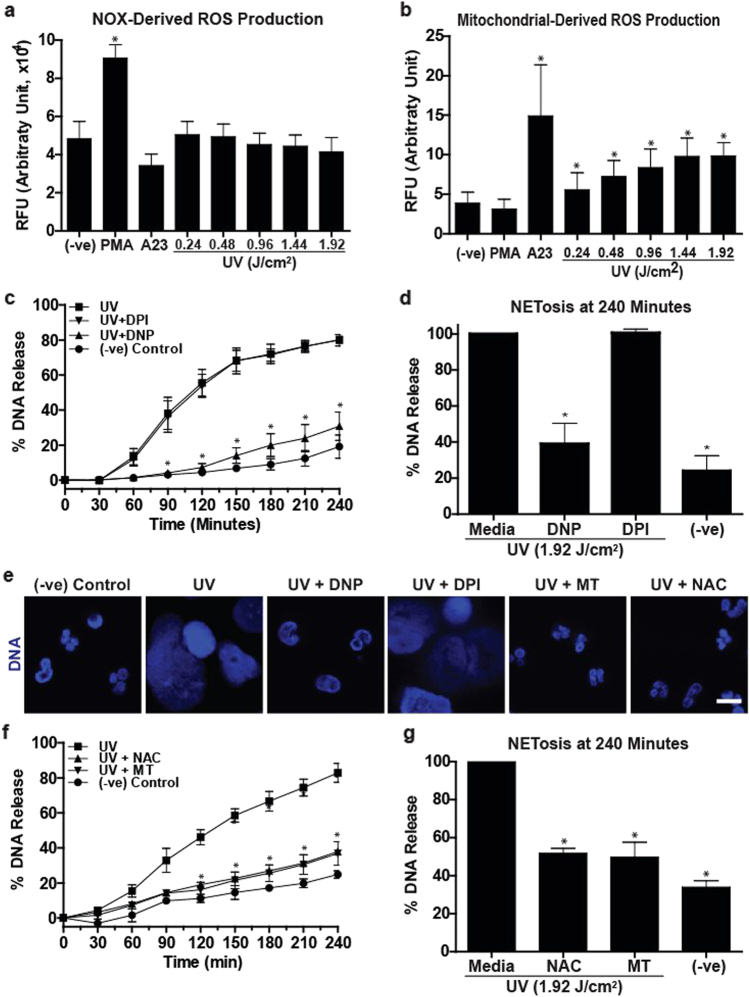
Mitochondrial but not NOX-mediated ROS production is involved in UV-induced NETosis. **a** NOX-derived ROS production was measured using the DHR123 plate reader assay. Cells were treated with PMA (25 nM), A23 (4 µM) or increasing doses of UV, and the fluorescence signals were recorded. None of the doses of UV induce NOX-derived ROS, similar to A23, except PMA that induces a significant amount of NOX-mediated ROS production (*n* = 3; error bars represent SEM; A23, A23187; *p < 0.05 for comparing with the negative control). **b** Mitochondrial ROS production was measured using the MitoSOX plate reader assay. Higher doses of UV induce mitochondrial-derived ROS, similar to A23 (4 µM), but unlike PMA (25 nM) that does not induce a significant amount of mitochondrial ROS production (*n* = 3; error bars represent SEM; A23, A23187; *p < 0.05 for comparing with the negative control). **c**,** d** DNA release following UV (1.92 J/cm^2^) treatment was measured using the SYTOX Green plate reader assay (*n* = 3). Cells were preincubated either with DPI (1 µM) or DNP (750 µM) for 1 h prior to UV treatment. DNP, but not DPI, significantly inhibits UV-induced NETosis. **e** Neutrophils were incubated with NAC (3 mM), MitoTempo (100 µM), DPI (1 µM) or DNP (750 µM) for 1 h and then treated with UV (1.92 J/cm^2^) and incubated for 240 min without SYTOX green. Cells were stained for DNA (DAPI, blue). Fluorescence imaging shows that NAC, MitoTempo and DNP, but not DPI, inhibit UV-induced NETosis. Images are representative of three independent experiments (scale bar, 10 μm). **f**,** g** DNA release following UV (1.92 J/cm^2^) treatment was measured using the SYTOX Green plate reader assay (*n* = 3). Cells were incubated with NAC (3 mM) or MitoTempo (100 µM) for 1 h prior to UV treatment. NAC and MitoTempo significantly inhibit UV-induced NETosis. Error bars on graphs represent SEM. MT, MitoTempo; *p < 0.05 for comparing UV treatment alone to UV treatment with inhibitors

ROS production is important for both NOX-dependent and -independent NETosis; however, NOX-mediated and mitochondria-mediated ROS production are essential for NOX-dependent and NOX-independent NETosis, respectively^18,33^. To determine the source of ROS production during UV-induced NETosis, we studied both types of ROS. hypochlorous acid (HOCl) produced during NOX-dependent NETosis is known to oxidise non-fluorescent DHR123 molecules to green fluorescent R123 molecules^34^. DHR123 plate reader assays showed that PMA, but not UV exposure, induced NOX-dependent ROS production (Fig. 3a). Next, we determined the mitochondrial ROS production using the non-fluorescent MitoSOX, a mitochondria-specific dye that fluoresces red upon oxidation. MitoSOX plate reader assays showed that UV exposure dose-dependently induced mitochondrial ROS production (Fig. 3b). Therefore, during NETosis both A23 and UV induce ROS of mitochondrial, but not of NOX in origin (Fig. 3).

To confirm the importance of NOX and mitochondria in UV-induced NETosis, we used a NOX inhibitor diphenyleneiodonium (DPI) and a mitochondrial uncoupler 2,4-dinitrophenol (DNP) in the SYTOX Green plate reader assays and immunocytochemistry experiments. Cells were incubated either with DPI (1 µM) or DNP (750 µM) or buffer for 60 min prior to exposure to UV. Neutrophils exposed to PMA and A23 were used as additional controls. DNP significantly decreased UV-induced NETosis while DPI failed to do so in SYTOX Green plate reader assay (Fig. 3c, d) and in immunocytochemistry analyses by confocal microscopy (Fig. 3e). These sets of data further confirm that UV induced NETosis via a mitochondria-mediated NOX-independent, but not NOX-dependent, pathway.

To directly show the importance of mitochondrial ROS in UV-induced NETosis, we used the general ROS scavenger *N*-acetyl-cysteine (NAC), and the mitochondrial-specific ROS scavenger MitoTempo (MT). Cells were incubated with NAC (3 mM) or MT (100 µM) or buffer for 60 min prior to exposure to UV. NAC and MT significantly inhibited UV-induced NETosis as determined by confocal imaging and SYTOX Green assays (Fig. 3e–g). These data directly show that mitochondrial ROS is necessary for UV-induced NETosis. Taken together (Fig. 3), UV induces a form of NOX-independent NETosis, which requires mitochondrial ROS.

### p38 MAPK activation is required for UV-induced NETosis

**Fig. 4:**
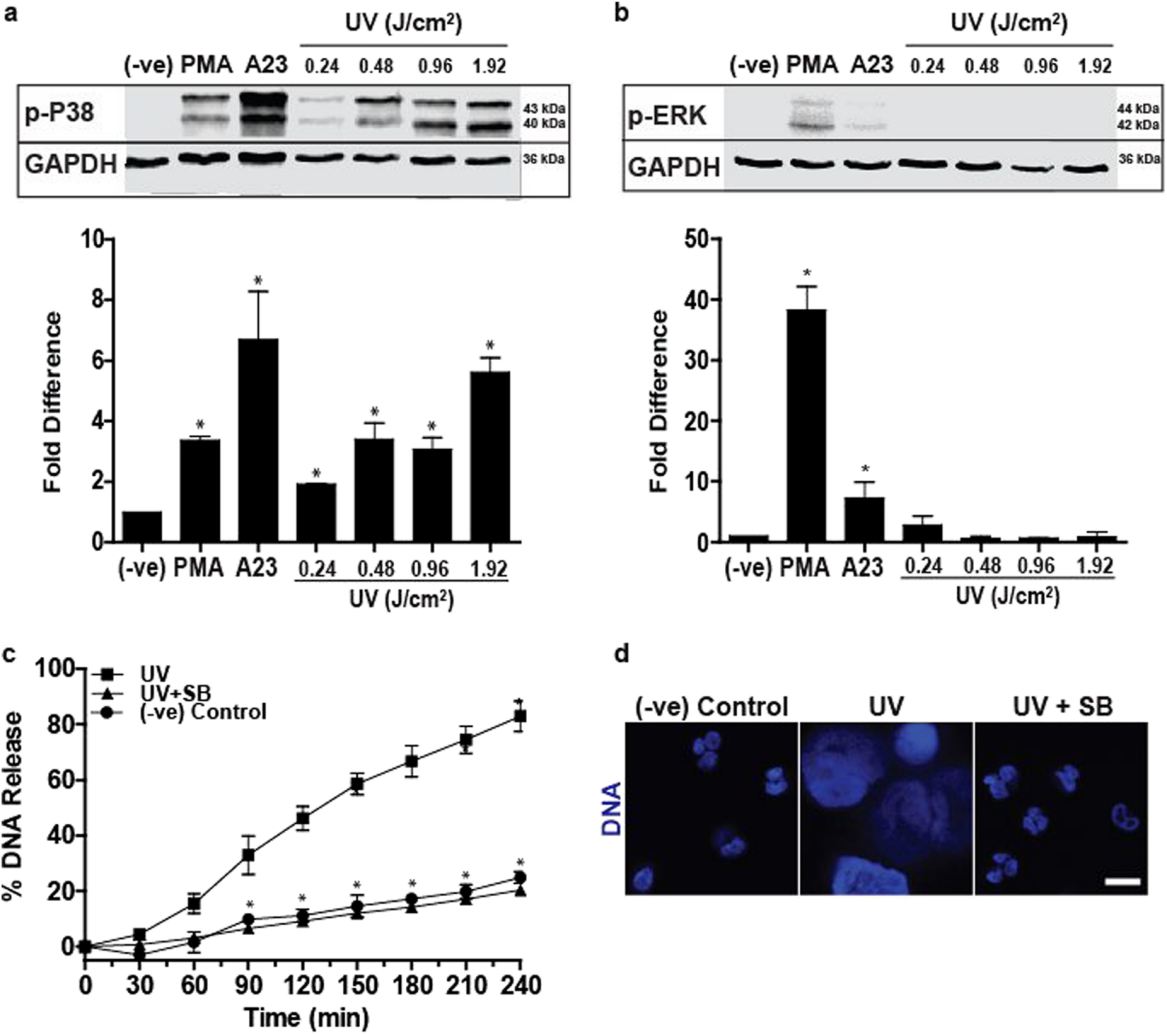
Increasing doses of UV increase the activation of p38, but not ERK kinase, and p38 activation is necessary for UV-induced NETosis. **a** Human neutrophils were collected 1 h after activation with PMA (25 nM), A23 (4 µM) or increasing doses of UV for western blot analysis. Immunoblots show that increase in UV dose increases p38 activation (determined by detecting phosphorylated p38 kinase). Densitometry analysis confirms the significant increase in p38 activation following UV treatment (*n* = 3). **b** Immunoblots show that ERK is not activated (determined by detecting phosphorylated ERK kinase) during UV irradiation. Densitometry analysis confirm the lack of ERK activation following UV treatment (*n* = 3). **c**,** d** Cells were incubated with p38 inhibitor SB203580 (20 µM) for 1 h prior to UV (1.92 J/cm^2^) treatment, and analysed with SYTOX Green assay. **d** Neutrophils were incubated with SB203580 (20 µM) for 1 h and then treated with UV (1.92 J/cm^2^) and incubated for 240 min. Cells were stained for DNA (DAPI, blue). Fluorescence imaging shows that SB203580 inhibits UV-induced NETosis (scale bar, 10 μm). Images are representative of three independent experiments. For all the graphs: error bars represent SEM; A23, A23187; SB, SB303580; *p < 0.05 for comparing UV treatment alone to UV treatment with inhibitors

Activation of specific groups of kinases is important for NETosis^18^. MAPK such as ERK is highly activated during NOX-dependent NETosis, whereas p38 is activated in both NOX-dependent and -independent NETosis^18^. Hence, we examined the activation status of ERK and p38 in the context of UV-induced NETosis. Neutrophils exposed to PMA and A23 were used as controls for NOX-dependent and NOX-independent NETosis, respectively. Immunoblot results revealed that p38, but not ERK, was activated in UV-induced NETosis (Fig. 4a, b). The p38 inhibitor SB203580 (20 µM) was then used for confirming the importance of p38 in UV-induced NETosis. SB203580 or buffer was incubated with cells for 60 min prior to exposure to UV and the effect of p38 inhibition was measured using the SYTOX Green plate reader assay and confocal imaging. SB203580 decreased UV-induced DNA decondensation and NETosis (Fig. 4c, d). Therefore, activation of MAPK p38 is necessary for UV-induced NETosis.

### UV-induced NETosis differs from calcium-induced NOX-independent NETosis

**Fig. 5:**
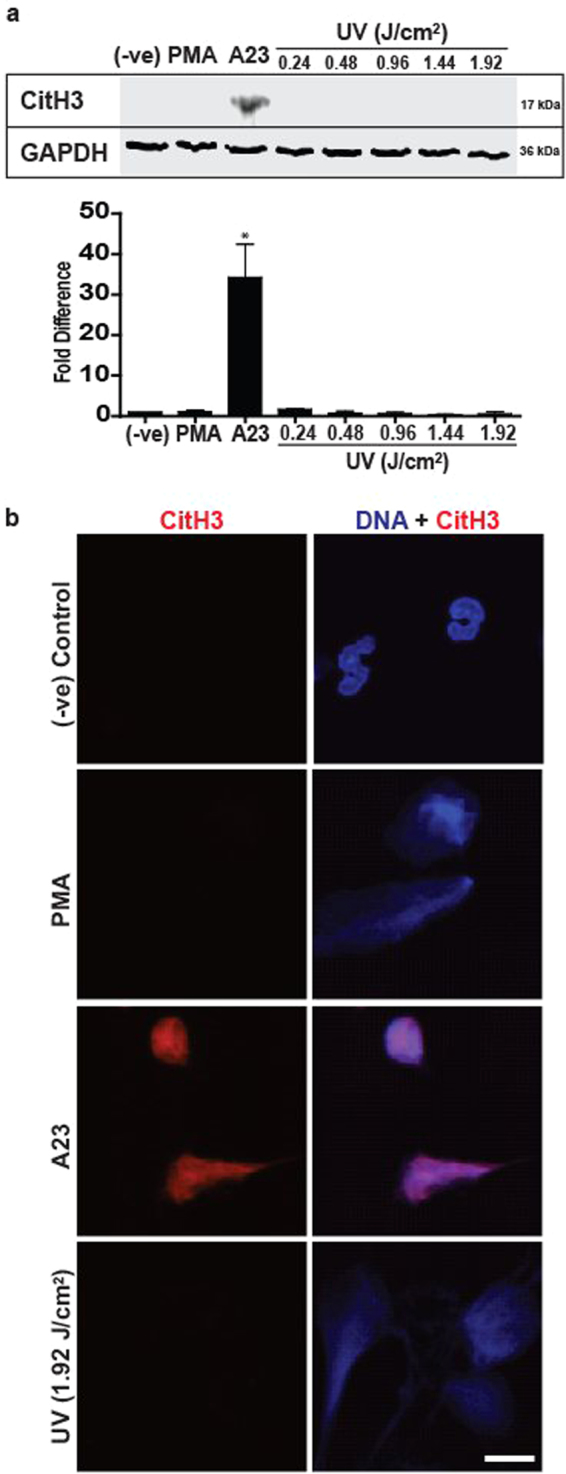
UV-induced NETosis does not induce citrullination of histones. **a** Human neutrophils were collected for western blot analysis 3 h after activation with PMA (25 nM), A23 (4 µM) or increasing doses of UV. Immunoblots show that citrullination of histones occurs in A23, but not in PMA or the UV conditions. Densitometry analysis confirm the lack of citrullination of histones following UV treatment (*n* = 3; error bars represent SEM; A23, A23187; *p < 0.05 for comparing to negative control). **b** Neutrophils were treated with PMA (25 nM), A23 (4 µM) or UV (1.92 J/cm^2^) and incubated for 180 min. Cells were fixed and stained for DNA (DAPI; blue) and citrullinated histones 3 (red). Imaging confirms that citrullination of histones is not found in cells undergoing UV-induced NETosis (scale bar, 10 μm). Images are representative of three independent experiments. See low-magnification images in Fig. S5

A hallmark of calcium-mediated NOX-independent NETosis is the citrullination of histones^25^. Since UV-induced NETosis is a form of NOX-independent NETosis, we examined whether it also induces citrullination of histones. Western blotting revealed that while stimulating neutrophils with A23 induced high levels of citrullination of histones, PMA treatment and UV irradiation did not generate detectable levels of citrullination of histones (Fig. 5a). This was further confirmed by immunocytochemistry. Images showed that A23 induced copious amounts of citrullinated histones (red), while PMA and UV irradiation failed to induce noticeable amounts of CitH3 (Fig. 5b, S5; 180 min time point in which CitH3 is clearly identifiable). Thus, while UV-induced NETosis is a NOX-independent form of NETosis, it differs from calcium-mediated NOX-independent NETosis.

### Transcriptional firing is necessary for UV-induced NETosis

**Fig. 6:**
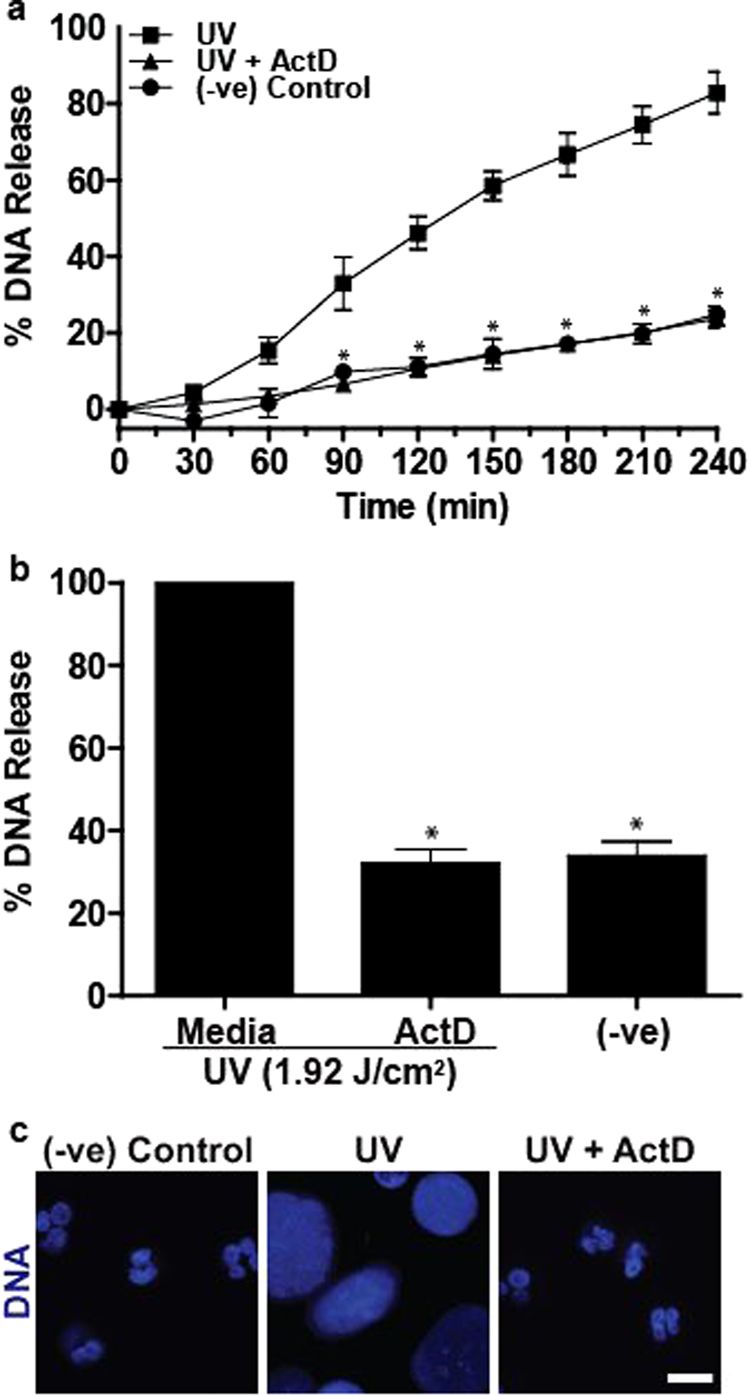
Transcriptional firing is required for UV-induced NETosis. **a**,** b** DNA release following UV (1.92 J/cm^2^) treatment was measured using the SYTOX Green plate reader assay (*n* = 3). Cells were incubated with Actinomycin D (10 µM) for 1 h prior to UV treatment. Actinomycin D significantly inhibits UV-induced NETosis. **c** Neutrophils were incubated with Actinomycin D (10 µM) for 1 h and then treated with UV (1.92 J/cm^2^) and incubated for 240 min. Cells were stained for DNA (DAPI; blue). Fluorescence imaging shows that Actinomycin D inhibits UV-induced NETosis (scale bar, 10 μm). Images are representative of three independent experiments. For all the graphs: error bars represent SEM. ActD, Actinomycin D; *p < 0.05 for comparing UV treatment alone to UV treatment with Actinomycin D

Transcriptional firing, a result of transcription factor activity initiated by kinase cascades, has been implicated in driving both NOX-dependent and -independent NETosis^24^. Because p38, a kinase responsible for transcription activation of many genes, is activated during UV-induced NETosis, we tested whether blocking transcription would inhibit NETosis. A commonly used transcription inhibitor Actinomycin D (ActD) inhibited UV-induced NETosis as determined by SYTOX Green assay (Fig. 6a, b) and confocal imaging of nuclear morphology (Fig. 6c). Therefore, UV-induced NOX-independent NETosis also uses transcriptional firing for DNA decondensation required for NETosis.

### UV-induced NETosis differs from necrosis, apoptosis and necroptosis

**Fig. 7:**
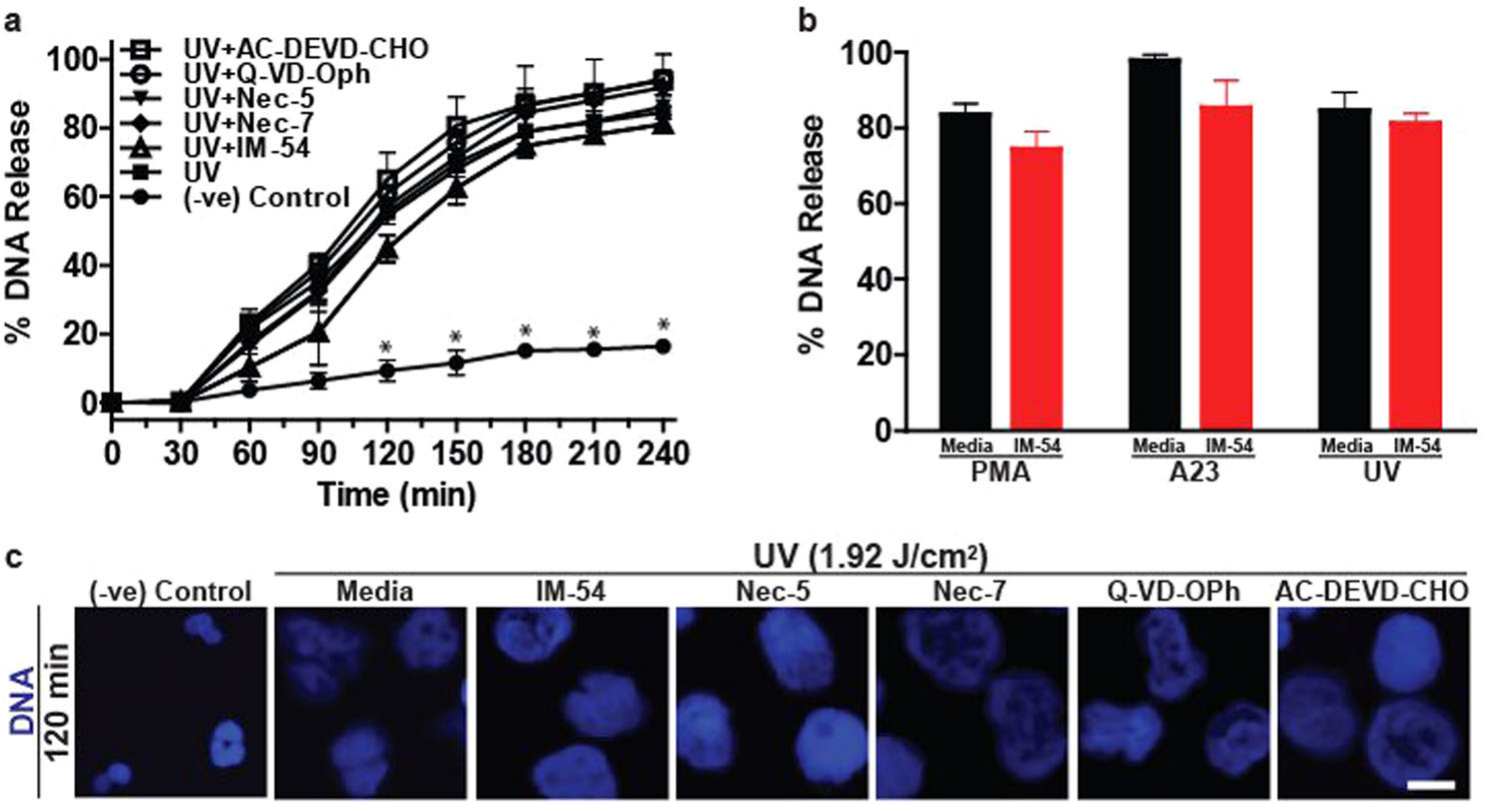
UV-induced NETosis differs from UV-induced apoptosis, necrosis and necroptosis. **a**,** b** DNA release following UV (1.92 J/cm^2^) treatment was measured using the SYTOX Green plate reader assay (*n* = 3). Cells were incubated with IM-54 (10 µM), necrostatin-5 (10 µM), necrostatin-7 (10 µM), QD-VD-Oph (10 µM) or AC-DEVD-CHO (10 µM) for 1 h prior to UV treatment. None of the inhibitors significantly inhibit UV-induced NETosis. **c** Experiments were conducted as above, except that SYTOX Green was omitted from the assay, and samples were incubated for 120 min. Cells were stained for DNA (DAPI; blue). Fluorescence imaging confirms that none of the inhibitors suppress UV-induced NETosis (scale bar, 10 μm). Images are representative of three independent experiments. Error bars represent SEM; *p < 0.05 for comparing UV treatment alone to UV treatment with inhibitors. See low-magnification images in Fig. S6

To differentiate the UV-induced NETosis from other common forms of programme cell deaths such as necrosis, apoptosis and necroptosis, we used pathway-specific inhibitors. The effects of the inhibitors of necrosis (IM-54), necroptosis (necrostatin-5, necrostatin-7) and apoptosis (QD-VD-Oph, AC-DEVD-CHO) were studied on UV-induced NETosis. Based on the concentrations necessary to inhibit specific pathways, cells were incubated with IM-54 (10 µM^35^), necrostatin-5 (10 µM^35^), necrostatin-7 (10 µM^36^), QD-VD-Oph (10 µM^37^) and AC-DEVD-CHO (10 µM^38,39^) for 60 min prior to exposure to UV. SYTOX Green assays showed that none of the apoptosis and necroptosis inhibitors affected UV-induced NETosis (Fig. 7a). IM-54 caused a small, statistically insignificant decrease in UV-induced NETosis (Fig. 7a). Similar results were observed in the PMA- and A23-treated cells (Fig. 7b). DNA decondensation examined by DAPI staining and confocal microscopy confirmed the SYTOX Green plate reader assay data. Imaging showed that cells treated with the inhibitors, including IM-54, all had completely decondensed chromatin, confirming that the inhibitors did not suppress UV-induced NETosis (Fig. 7c, S6). Apoptotic cell death was activated during UV-induced neutrophil death; however, caspase inhibition did not affect UV-induced NETosis. Therefore, NETotic events had overridden apoptotic events. These studies (Fig. 7) indicate that high-dose UV-induced neutrophil death is neither necrosis nor necroptosis, but a novel form of apoptosis/NETosis.

## Discussion

**Fig. 8:**
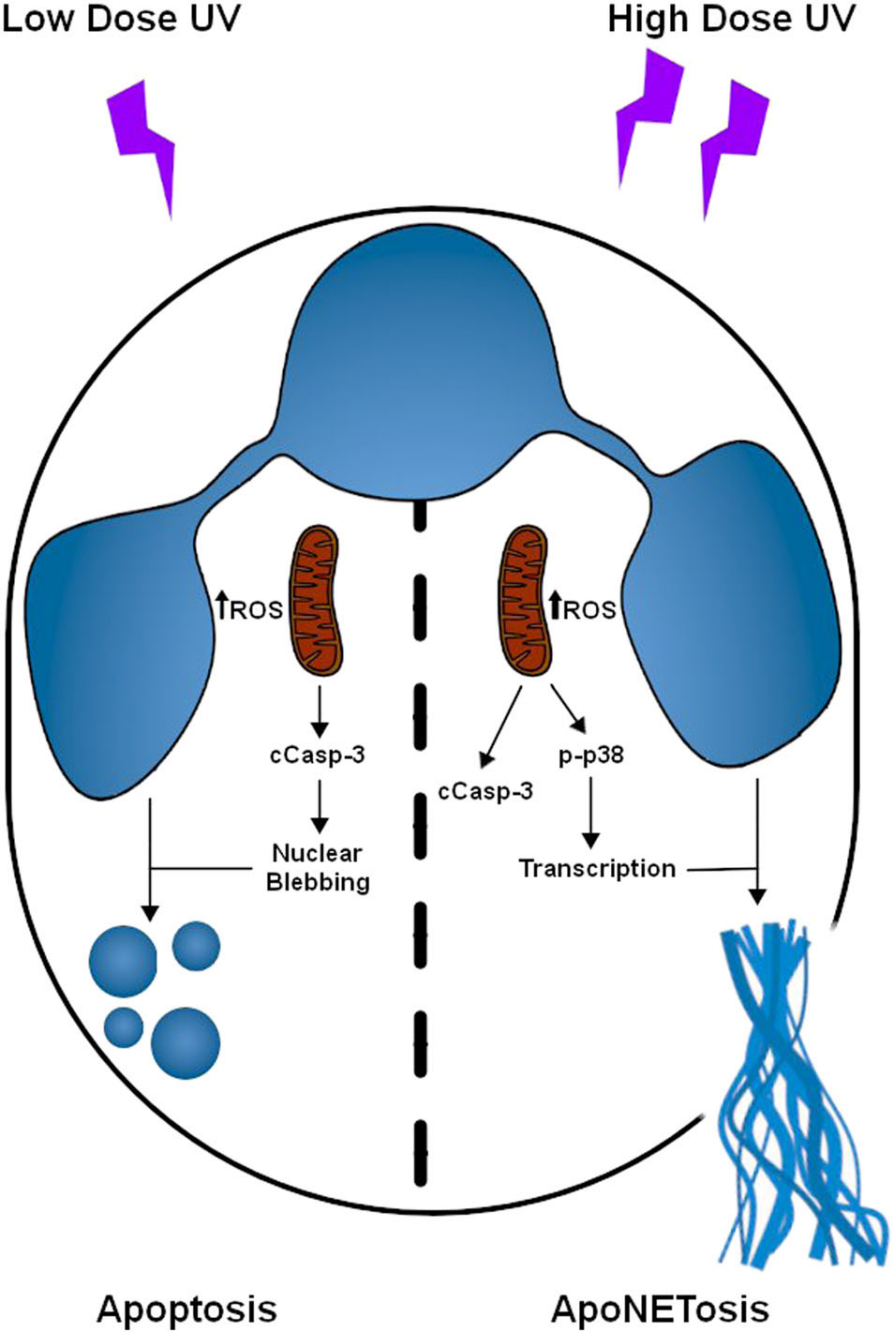
A model showing the key steps of UV-induced apoptosis and ApoNETosis. Low-dose UV irradiation induces small amounts of mitochondrial ROS, and subsequent caspase cascade activation and classical intrinsic pathway of apoptosis. By contrast, increasing doses of UV irradiation lead to increased production of mitochondrial ROS, caspase cascade activation, p38 activation, transcriptional firing and NETosis. Under ApoNETosis conditions, although both apoptosis and NOX-independent NETosis occur simultaneously, NETotic events predominate apoptotic events. cCasp-3, cleaved caspase 3. Thickness of the upwards arrow indicates the amounts of ROS

UV is routinely used for inducing apoptosis^28^; however, whether UV induces NETosis is unknown. Our present studies indicate that while low-dose UV induces apoptosis, high doses of UV irradiation induce NETosis. We have identified that UV-induced NETosis is rapid, and follows the kinetics typical for NOX-independent NETosis. With increasing doses of UV irradiation, apoptotic pathway leading to the activation of executioner caspase 3 (cleaved caspase 3) is increased. At the same time, an increase in UV dose increases DNA decondensation and NET formation, but not classical apoptotic changes such as DNA condensation. This increased NETosis is associated with mitochondrial ROS production and p38 MAPK activation. Inhibitor studies established that UV-induced NETosis is a NOX-independent process; however, unlike calcium ionophore-induced NETosis, UV-induced NETosis does not induce citrullination of histone. Nevertheless, similar to classical NOX-dependent and calcium ionophore-induced NOX-independent NETosis, UV-induced NETosis also requires transcriptional firing for DNA decondensation. Finally, cell death-specific inhibitor studies indicate that UV-induced NETosis is unique, and it is not apoptosis, necrosis or necroptosis. In this form of cell death, both apoptotic and NETotic pathway steps are operative; however, NETotic events overrides the apoptotic events. Therefore, we name this novel form of UV-induced NETosis as ApoNETosis (Fig. 8).

UV has been previously shown to induce apoptosis via the intrinsic pathway in many cell types including neutrophils^30^. UV-induced apoptosis involves mitochondrial ROS generation^40^ and the release of cytochrome *c* from mitochondria, and eventual cleavage and activation of caspase 3^30^. Among other cellular changes, a remarkable event that occurs during apoptosis is the condensation of nuclei^41^. As expected, neutrophils exposed to low-dose UV irradiation underwent apoptosis (Figs. 1 and 2). When the UV dose is increased, caspase 3 cleavage is also increased (Fig. 2c). Based on the conventional wisdom, increased caspase 3 activation would be expected to increase apoptosis^31^. Unexpectedly, increased activation of caspase 3 did not lead to increase in apoptotic nuclei; instead, neutrophil nuclei become decondensed (Fig. 2d). To confirm whether these cells are undergoing apoptosis^30^ or NETosis^7^, we immunolabelled cells with cleaved caspase 3 and MPO. MPO is present in the cytoplasmic granules of neutrophils^42^. During NETosis MPO migrates into the nuclei and decorates decondensed chromatin and is released as part of the NETs^7^. This NET marker clearly colocalises to decondensed chromatin inside the nuclei and extracellular DNA, confirming that increasing doses of UV irradiation increase NETosis (i.e., granular proteins entering the nuclei, and then coating chromatin before the release of these complexes as NETs^7^). Therefore, UV induces typical NETs with MPO, but not MPO binds to the released chromatin. Examination of cleaved caspase 3 indicates that the active caspase is distributed throughout these NETotic cells (Fig. 2b). Therefore, both apoptotic and NETotic programmes simultaneously take place in every cell at high-dose UV irradiation. No such type of cell death has been reported previously^15,43,44^. Therefore, we further examined different molecular steps involved in this novel type of NETosis.

During NOX-dependent NETosis (e.g., induced by PMA, lipopolysaccharide, bacteria), NOX is activated to generate ROS^33^, specific MAPKs such as ERK^18^, p38^18^ and JNK^23^ are differentially activated^18^ and, as the last step, transcriptional firing occurs to facilitate chromatin decondensation and NET release^24^. By contrast, during NOX-independent NETosis (e.g., induced by calcium ionophores, uric acid and other crystals) NOX is not activated^18,45^. Instead of NOX-mediated ROS, mitochondrial ROS predominates during NOX-independent NETosis^18^. Our present data show that UV-induced NETosis is not dependent on NOX; NOX inhibitor DPI^22^ did not affect UV-induced NETosis and UV irradiation of neutrophils did not generate ROS that is detectable by DHR123 (Fig. 3a). By contrast, UV irradiation dose-dependently increased mitochondrial ROS (Fig. 3b), and the mitochondrial uncoupler DNP^18^ and mitochondrial ROS scavenger MitoTempo^46^ inhibited UV-induced NETosis (Fig. 3e). Therefore, mitochondrial ROS requirement of UV-mediated NETosis is similar to that of A23-mediated NOX-independent NETosis^18^. During calcium ionophore-induced NETosis, PAD4 is highly activated and transported into the nuclei to citrullinate histones^47^. Since A23 increases intracellular calcium levels^48^, citrullination of histones occurs in A23-^18,19^, but not UV-induced NETosis (Fig. 5).

Several studies have established that kinase activation is important for both NOX-dependent and NOX-independent NETosis^18,23^. Furthermore, we and others have shown that transcription, but not translation and new protein synthesis, are necessary for NETosis^24,49^. Therefore, it is unlikely that the expression of pro-survival Bcl-2 family protein Mcl-1^50^ is altered within a short time during NETosis. UV irradiation activates p38, but not ERK, and the suppression of p38 inhibits NETosis (Fig. 4). Therefore, p38 activation is essential for UV-induced NETosis. Activated p38 is known to activate several transcription factors^51^. Inhibition of transcription with ActD^24^ inhibits UV-induced NETosis, indicating that transcriptional firing is important for this form of NETosis as well (Fig. 6).

Neutrophils can die by various forms of cell death^28,29^. Therefore, to verify whether UV-induced NETosis is related to other forms of cell death, we used pathway-specific inhibitors^35,37–39^. Inhibitors of apoptosis, necrosis and necroptosis do not inhibit UV-induced NETosis (Fig. 7). Therefore, high-dose UV induces NETosis de novo while the apoptosis pathway is active. Caspase 3 is activated, yet apoptosis inhibitors do not inhibit UV-induced NETosis. Therefore, terminal step(s) of apoptosis such as the activation of caspase 3-activated DNAses that fragment chromatin DNA may be disabled in this form of NETosis. During NETosis, the granular proteases (e.g., elastases, proteinase 3) and MPO that enters nucleus cleave and modify proteins^7^. Therefore, this is a plausible mechanism that disables the later steps of apoptosis and allows the progression of NETosis. Previous studies showed that apoptosis is distinct from NETosis^52^, and apoptosis (e.g., caspase 3 cleavage) did not occur during NETosis^21,53^. Although initial studies implicated autophagy as an important component of NETosis^21^, recent studies indicate that autophagy is not necessary for NET formation, and necrosis is different from NET formation^1^. Furthermore, UV-induced NETosis involves nuclear DNA, and is not a vital form of NETosis. Our present studies show that UV irradiation, a typical inducer of apoptosis, induces both apoptosis and NETosis simultaneously in the same neutrophil. The molecular understanding of this new form of NETosis, ApoNETosis, would be helpful for understanding the various steps of different types of NETosis.

UV is often used as a valuable non-chemical method to study cell death^28,29^. UV-irradiated apoptotic cells, rather than chemically induced apoptotic cells, are often used for efferocytosis studies because UV irradiation avoids the complications of cytotoxic chemicals present together with dead cells^54^. UV-induced NETosis could be used for obtaining specific types of NETs (without CitH3) for phagocyte-mediated NET clearance studies^55^. UV-induced NETosis model could also be used for studying the effect of UV light exposure of the skin, which has vasculature^56^. UV is a key agent that induces skin damage^57^, and excess UV exposure often results in perivascular inflammation^56^. Certain groups of patients (e.g., with vitiligo and psoriasis) are subjected to peripheral exposure to UV light as part of their treatment. The doses of UV used for these treatments^58–60^ are greater than the ones used in our study. Therefore, delineating the molecular steps involved in UV-mediated NETosis, or ApoNETosis, is useful for understanding the inflammation and treatment options for specific disease states.

## Methods

### Ethical clearance

The protocol was approved by the Hospital for Sick Children ethics committee. All methods were performed in adherence with the set guidelines and regulations. Subjects signed informed consent forms.

### Neutrophil isolation from human peripheral blood

Peripheral blood was drawn from healthy donors and placed in K2 EDTA blood collection tubes. Neutrophils were isolated using PolymorphPrep (Axis-Shield) as previously published^6^. Slight changes to the manufacturer’s neutrophil isolation protocol were made. A hypotonic solution of 0.2% (w/v) NaCl was used for lysing the red blood cells. The solution was made isotonic and buffered by adding an equal volume of 1.6% (w/v) NaCl solution with Hepes buffer (20 mM, pH 7.2). The cells were washed with a solution of 0.85% (w/v) NaCl and Hepes (10 mM, pH 7.2) twice. Neutrophils were resuspended in RPMI medium (Invitrogen) containing Hepes buffer (10 mM, pH 7.2) for experiments.

### Inducing NETosis and apoptosis with UV light

UV irradiation of cells was performed using a Stratalinker 2400 (Stratagene) machine. Cells were irradiated with 0.24 to 1.92 J/cm^2^ of UVC light to induce apoptosis and NETosis, respectively. Different UV doses within this range were used for identifying the relative contributions of NETosis and apoptosis in neutrophils.

### SYTOX Green plate reader assay for NETosis analysis

SYTOX Green was added to cells (5 × 10^5^ cells per ml) at a concentration of 1:1000 (5 μM; ThermoFisher Scientific). The cells were seeded on a 96-well plate and then incubated with various inhibitors for 1 h at 37 °C. The cells were then activated with either media control (−ve control), different dosage of UV or known NOX-dependent (25 nM PMA) and -independent agonists (4 µM A23). The NETosis kinetics was assessed by measuring the fluorescence of SYTOX Green-DNA interactions using POLARstar OMEGA fluorescence plate reader (BMG Labtech; excitation = 485 nm, emission = 525 nm) every 30 min for 240 min. Maximal levels of DNA release were determined by lysing cells with 1% (v/v) Triton X-100. NETosis index was determined by dividing the SYTOX Green reading of each condition by the reading of 1% (v/v) Triton X-100-treated cells taken at 240 min.

### DHR123 plate reader assay (NOX-derived ROS Analysis)

Cells at a concentration of 1 × 10^6^ cells per ml were seeded on a 96-well plate. Cells were UV irradiated, and then 25 µM DHR123 was added to the wells. After cell activation, DHR123 oxidation to florescent R123 was measured using POLARstar OMEGA fluorescence plate reader (BMG Labtech; excitation = 485 nm, emission = 525 nm) after 240 min.

### MitoSOX plate reader assay (Mitochondrial ROS analysis)

Cells at a concentration of 1 × 10^6^ cells per ml were seeded on a 96-well plate. Cells were UV irradiated, and then 5 µM MitoSOX was added to the wells. Directly following cell activation, florescence of MitoSOX oxidation was measured using POLARstar OMEGA fluorescence plate reader (BMG Labtech; excitation = 510 nm, emission = 580 nm) after 30 min.

### Confocal imaging

Cells at a concentration of 1 × 10^6^ cells per ml were plated on a 96-well plate, and incubated with inhibitors for 1 h at 37 °C. Following induction of NETosis, reaction proceeded for an allotted amount of time at 37 °C before being terminated with 4% (w/v) paraformaldehyde (Sigma Aldrich) overnight. Cells were permeabilised with 0.1% Triton X-100 for 15 min and then blocked with 2.5% (w/v) bovine serum albumin (BSA) in phosphate-buffered saline (PBS) for 1 h. MPO was probed for using mouse anti-myeloperoxidase antibody (ab25989, Abcam) at a 1:500 dilution. Cleaved-caspase 3 was probed by rabbit anti-cleaved-caspase 3 antibody (ASP175, Cell Signalling) at a 1:500 dilution. Citrullinated histone was probed by rabbit anti-histone H3 (citrulline R2+R8+R17) antibody (ab5103, Abcam) at a 1:500 dilution. DAPI (10 μM; ThermoFisher Scientific) at 1:333 dilution was used for visualising DNA. Imaging was done using Olympus IX81 inverted fluorescence microscope with a Hamamatsu C9100-13 back-thinned EM-CCD camera and Yokogawa CSU ×1 spinning disk confocal scan head.

### Western blotting

For lysates, 5 × 10^5^ cells were placed in Eppendorf tubes in a volume of 40 μl. The cells were then incubated with various inhibitors for 1 h at 37 °C. Following induction of NETosis, incubation proceeded for 1 h at 37 °C. Samples were lysed with the addition of 10 μl of lysis buffer containing complete protease inhibitor mixture (Roche) supplemented with NaVO_3_ (5 mM), leupeptin (125 µM), pepstatin (125 μM), aprotinin (125 µM), NaF (125 mM), levamisole (5 mM), freshly prepared phenylmethylsulfonyl fluoride (5 mM), and 0.5% (w/v) Triton X-100. Samples were then sonicated thrice for 3 min using an aquasonic sonicator (VWR, model 50D) at maximal settings. Loading buffer (5×; 10 μl) containing Tris-HCl (125 mM, pH 6.8), 6% (w/v) SDS, 8% (v/v) β-mercaptoethanol, 18% (v/v) glycerol, 5 mM EDTA, 5 mM EGTA, leupeptin (10 μg/ml), pepstatin (10 μg/ml), aprotinin (10 μg/ml), NaF (10 mM), NaVO3 (5 mM) and levamisole (1 mM) was added to samples. Samples were then heated at 95 °C for 10 min on a heat block (Eppendorf). The samples were size-fractionated on a 12% (w/v) resolving and 5% (w/v) stacking bis-acrylamide gels for 25 min at 100 V and 30 min at 200 V. Using a wet transfer system, proteins were transferred from the gel onto a nitrocellulose membrane for 90 min. The membrane was blocked with 5% (w/v) BSA in PBS containing 0.05% (w/v) Tween-20 buffer for 1 h at room temperature. The antibodies used were anti-phospho-ERK1/2 (AW39R; EMD Millipore) at 1:500; anti-phospho-p38 MAPK (D3F9) XP (Cell Signaling) at 1:500; anti-cleaved caspase 3 (ASP175, Cell Signalling); and anti-histone H3 (citrulline R2+R8+R17) ChIP Grade (ab5103, Abcam). Secondary antibodies used were conjugated with horseradish peroxidase (HRP). HRP substrates used were enhanced chemiluminescent (ECL) reagents. Membranes were imaged using a Li-Cor Odyssey FC Imaging System. Anti-GAPDH (Santa Cruz) at 1:3333 was used for probing for the housekeeping protein GAPDH.

### Statistical analyses

Statistical analyses were performed using GraphPad Prism 7. One-way analysis of variance (ANOVA) with Dunnett and Tukey’s post tests, two-way ANOVA with Bonferroni post-test and Student’s* t*-test were performed as appropriate. Error bars in graphs represent ±SEM. A *p* value of less than 0.05 was considered to be statistically significant.

## Electronic supplementary material

Figure S1

Figure S2

Figure S3

Figure S4

Figure S5

Figure F6

Supplementary Figure Legends
