# Supplementary figures and images for: Two-in-one: UV radiation simultaneously induces apoptosis and NETosis
Source: Cell Death Discov. 2018 Apr 27;4:51. doi: 10.1038/s41420-018-0048-3 (PMC5919968; doi:10.1038/s41420-018-0048-3)

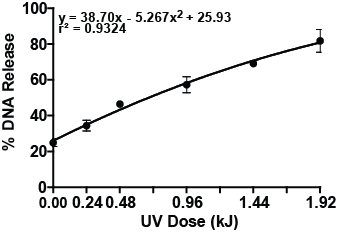

Supplement: Supplementary file 1 — Figure S1 [file 41420_2018_48_MOESM1_ESM.jpg]

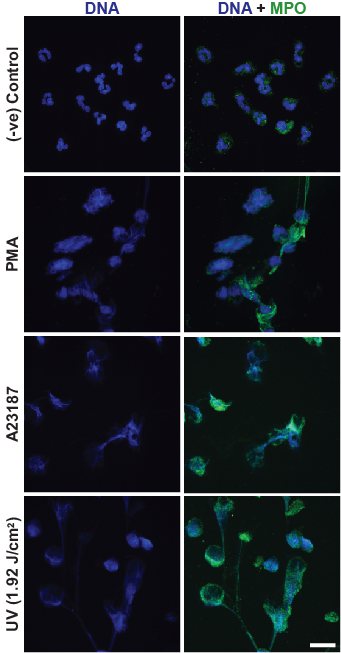

Supplement: Supplementary file 2 — Figure S2 [file 41420_2018_48_MOESM2_ESM.jpg]

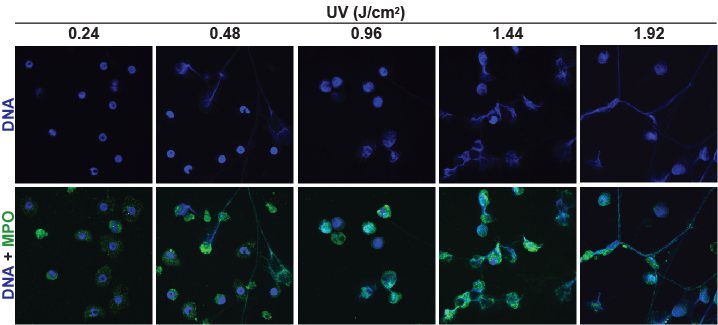

Supplement: Supplementary file 3 — Figure S3 [file 41420_2018_48_MOESM3_ESM.jpg]

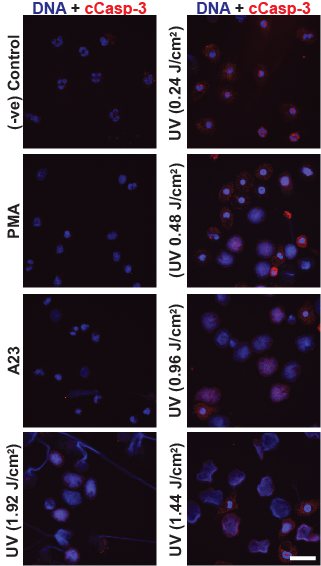

Supplement: Supplementary file 4 — Figure S4 [file 41420_2018_48_MOESM4_ESM.jpg]

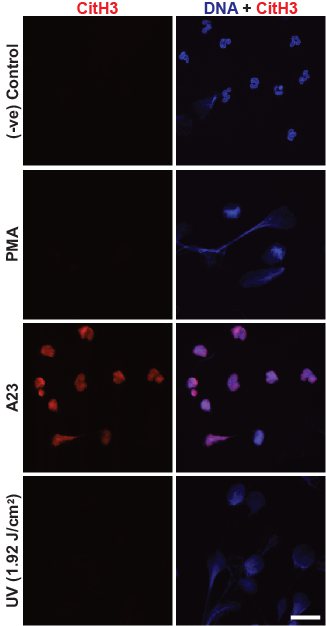

Supplement: Supplementary file 5 — Figure S5 [file 41420_2018_48_MOESM5_ESM.jpg]

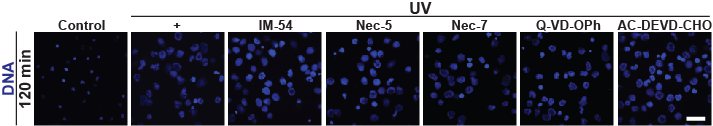

Supplement: Supplementary file 6 — Figure F6 [file 41420_2018_48_MOESM6_ESM.jpg]
