# Supplementary material for: Two-in-one: UV radiation simultaneously induces apoptosis and NETosis
Source: Cell Death Discov. 2018 Apr 27;4:51. doi: 10.1038/s41420-018-0048-3 (PMC5919968; doi:10.1038/s41420-018-0048-3)
Supplement: Supplementary file 7 — Supplementary Figure Legends [file 41420_2018_48_MOESM7_ESM.docx]

**Supplementary Figure Legends**

**Figure S1.** DNA release following treatment with increasing dose of UV was measured using the SYTOX Green plate reader assay (n = 3; error bars represent SEM; *p < 0.05). Increasing dose of UV results in increased NETosis. The dose response at 240-min time point follows a saturation curve indicating UV dose positively correlates with NETosis and cell death plateaus at high doses.

**Figure S2.** Confocal images at low magnification indicate that DNA released following UV exposure is colocalized with MPO (240-min time point; Blue, DNA (DAPI); Green, MPO; Scale bar, 20 μm). Images are representative of three independent experiments.

**Figure S3.** Confocal images at low magnification indicate that DNA release following UV treatment occurs in a dose dependent manner (240-min time point; Blue, DNA (DAPI); Green, MPO; Scale bar, 20 μm). Images are representative of three independent experiments.

**Figure S4.** Confocal images at low magnification indicate that cleaved caspase 3 is present in cells radiated with all doses of UV irradiation (120-min time point; Blue, DNA (DAPI); Red, cleaved caspase 3; Scale bar, 20 μm). Images are representative of three independent experiments.

**Figure S5.** Confocal images at low magnification show that A23187, but not UV, induces citrullination of histones (180-min time point; Blue, DNA (DAPI); Red, CitH3; Scale bar, 10 μm). Images are representative of three independent experiments.

**Figure S6.** Confocal images at low magnification show that IM-54, necrostatin-5, necrostatin-7, QD-VD-Oph and AC-DEVD-CHO fail to inhibit UV-induced NETosis (120-min time point; Blue, DNA (DAPI); Scale bar, 40 μm). Images are representative of three independent experiments.
